# Cytokinin facilitates the patterning of the adventitious root apical meristem from leaf cuttings

**DOI:** 10.1186/s43897-024-00091-6

**Published:** 2024-03-26

**Authors:** Ning Zhai, Beibei Sun, Shasha Wu, Feng Zhou, Yuling Jiao, Lin Xu

**Affiliations:** 1 National Key Laboratory of Plant Molecular Genetics, CAS Center for Excellence in Molecular Plant Sciences, Institute of Plant Physiology and Ecology, Chinese Academy of Sciences, 300 Fenglin Road, Shanghai, 200032 China; 2 Key Laboratory of Plant Carbon Capture, CAS, Shanghai, 200032 China; 3 College of Life Sciences, Shanghai Normal University, Shanghai, 200234 China; 4 School of Life Sciences and Peking-Tsinghua Center for Life Sciences, Peking University, Beijing, 100871 China

In cuttings, adventitious roots can be regenerated from detached or wounded plant organs, and this process is known as de novo root regeneration. In *Arabidopsis thaliana* leaf cuttings (Liu et al. 2014; Xu 2018), detached *A. thaliana* leaves are responsive to wound signals and many environmental stimuli, and then synthesize a certain level of auxin, which is transported to regeneration-competent cells (i.e., procambium and some vascular parenchyma cells near the wound site) to promote cell fate transitions for adventitious root organogenesis (Xu 2018). Auxin is the key hormone mediating the cell fate transitions from regeneration-competent cells to adventitious root apical meristem (adRAM) cells. In the first step of cell fate transition (i.e., priming), the auxin signaling pathway promotes the expression of *WUSCHEL-RELATED HOMEOBOX11* and *12* (*WOX11*/*12*), which lead to the conversion of regeneration-competent cells to adventitious root founder cells (Liu et al. 2014). In the second step (i.e., initiation), WOX11/12 and the auxin signaling pathway induce the expression of *LATERAL ORGAN BOUNDARIES DOMAIN16* (*LBD16*), *WOX5*/*7*, and *RGF1 INSENSITIVE1*/*2* (*RGI1*/*2*), which facilitate the transition of adventitious root founder cells to adventitious root primordium (adRP) cells via cell division (Liu et al. 2014; Zhang et al. 2023). In the third step (i.e., patterning), the adRP cells divide continuously with the patterning of different tissue domains to form the adRAM. A previous study that examined adventitious rooting from hypocotyls indicated that cytokinin might influence the patterning of the adRAM (Della Rovere et al. 2013). In the fourth step (i.e., emergence), the mature adventitious root tip is formed and grows out of the leaf explant. Although the role of auxin in this process has been extensively studied, our knowledge of the contribution of cytokinin to de novo root regeneration is still limited. In this study, we revealed that cytokinin is required for the patterning step that determines the tissue differentiation of the adRAM.

We first analyzed the role of cytokinin in adventitious rooting from detached *A. thaliana* leaves. Briefly, *A. thaliana* seedlings were grown on half-strength Murashige and Skoog medium at 22 °C with a 16-h light/8-h dark cycle. The first rosette leaf pair was cut from 12-day-old seedlings and cultured on B5 medium (Gamborg B5 basal medium with 0.5 g/L MES, 3% sucrose, and 0.8% agar, pH 5.7) to regenerate adventitious roots in darkness. Treating the detached leaves with the cytokinin biosynthesis inhibitor lovastatin (LOV) (Crowell & Salaz 1992) adversely affected rooting (Fig. 1A, B). Mutations in the cytokinin receptor-encoding *ARABIDOPSIS HISTIDINE KINASE* (*AHK*) genes (i.e., in the *ahk2-2 ahk3-3 ahk4-1* triple mutant) (Higuchi et al. 2004) or the cytokinin signaling-related transcription factor-encoding B-type *ARABIDOPSIS RESPONSE REGULATOR* (*ARR*) genes (i.e., in the *arr1-3 arr10-5 arr12-1* triple mutant) (Mason et al. 2005) resulted in the partially defective regeneration of adventitious roots from detached leaves (Bustillo-Avendaño et al. 2018) (Fig. 1C). Accordingly, cytokinin is likely involved in the adventitious rooting from detached leaves.

**Fig. 1:**
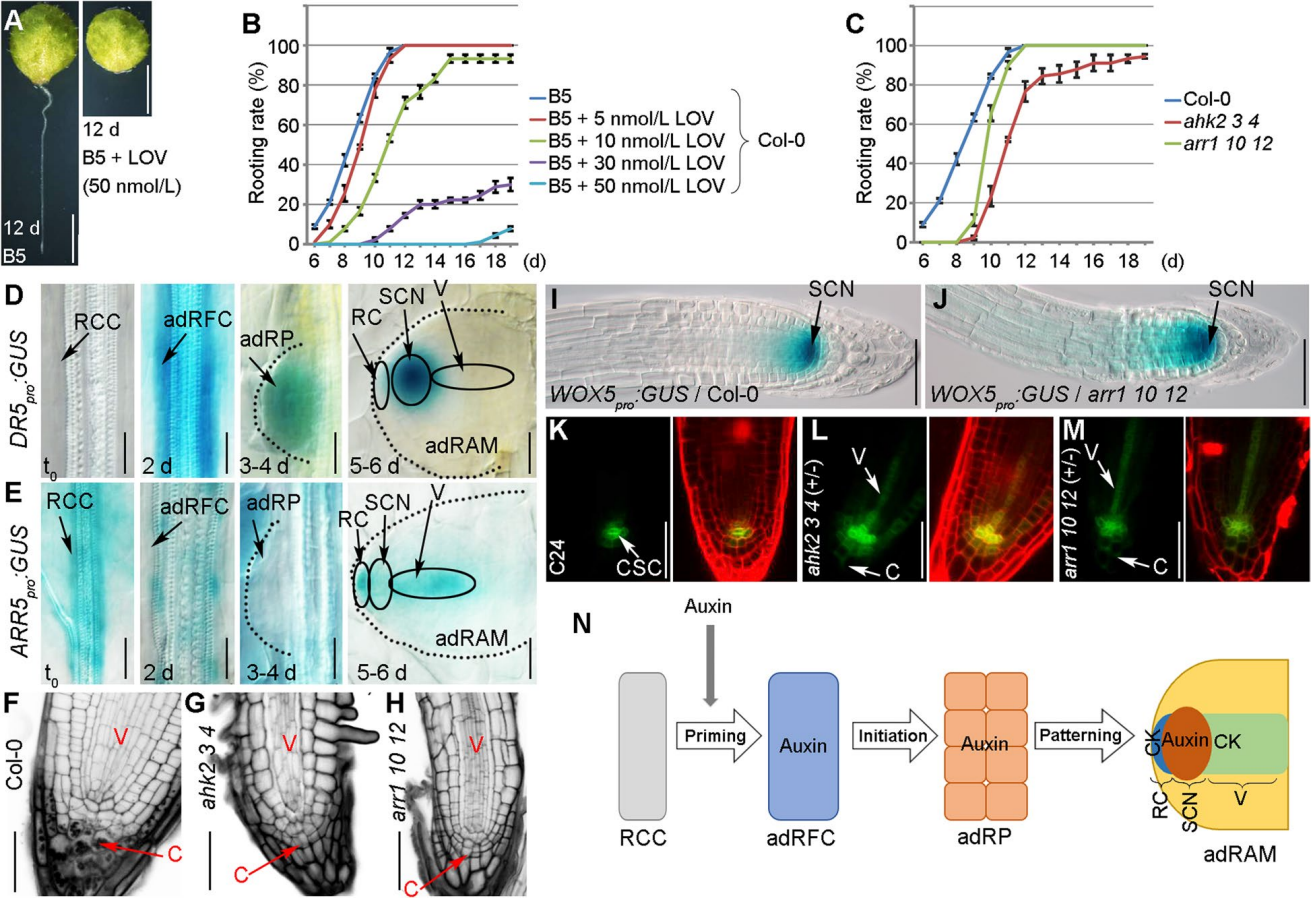
Roles of auxin and cytokinin in de novo root regeneration. **A**, **B** Phenotype (**A**) and adventitious rooting rate (**B**) of the detached Col-0 leaves treated with LOV. d, days. **C** Adventitious rooting rate of the detached leaves from Col-0, *ahk2-2 ahk3-3 ahk4-1* (*ahk2 3 4*), and *arr1-3 arr10-5 arr12-1* (*arr1 10 12*). Values shown are mean ± s.e.m. from three biological replicates (*n* = 30 in each replicate) (**B**, **C**). The experiment in (**C**) was performed together with the LOV treatment experiment in (**B**), therefore using the same control data, i.e. B5 in (**B**) and Col-0 in (**C**). **D**, **E** Expression patterns of *DR5*_*pro*_*:GUS* (**D**) and *ARR5*_*pro*_*:GUS* (**E**) in regeneration-competent cells at t_0_, adventitious root founder cells at 2 days, adRP at 3 to 4 days, and adRAM at 5 to 6 days. **F–H** Results of the mPS-PI staining of the adventitious root tips from Col-0 (**F**), *ahk2-2 ahk3-3 ahk4-1* (**G**), and *arr1-3 arr10-5 arr12-1* (**H**). Black dots in the Col-0 columella represent starch grains (**F**). **I**, **J** Expression pattern of *WOX5*_*pro*_*:GUS* in the adventitious root tips in the Col-0 (**I**) and *arr1-3 arr10-5 arr12-1* (**J**) genetic backgrounds. **K–M** Expression pattern of J2341 in the adventitious root tips in the C24 (**K**), *ahk2-2 ahk3-3 ahk4-1* (+ / −) (**L**), and *arr1-3 arr10-5 arr12-1* (+ / −) (**M**) genetic backgrounds. *AHK4-1* and *ARR12-1* are heterozygous. The left panels present the J2341 fluorescent signals, whereas the right panels present the J2341 and PI fluorescent signals. **N** Model of the effects of auxin and cytokinin during the formation of the adRAM. RCC, regeneration-competent cells; adRFC, adventitious root founder cells; adRP, adventitious root primordium; RC, root cap; SCN, stem cell niche; V, vasculature; C, columella; CSC, columella stem cells; SCN, stem cell niche; adRAM, adventitious root apical meristem; CK, cytokinin; + / − , heterozygous. Scale bars, 2 mm (**A**) and 50 μm (**D**–**M**)

We next examined auxin and cytokinin patterns during adventitious root organogenesis. In the auxin marker line transformed with *DR5*_*pro*_*:GUS* (Ulmasov et al. 1997), the GUS signal was almost undetectable in the regeneration-competent cells at time 0 (t_0_), whereas it was relatively strong in the adventitious root founder cells at approximately 2 days after the leaves were detached as well as in the adRP at approximately 3 to 4 days after the leaves were detached (Fig. 1D). Notably, in the adRAM, the signal was restricted to the stem cell niche and was absent in the root cap and vascular regions at approximately 5 to 6 days after the leaves were detached (Fig. 1D). In addition, LOV treatment could cause the ectopic GUS signal in the root cap region of the adRAM (Supplementary Fig. S1A).

The cytokinin signaling reporter line *ARR5*_*pro*_*:GUS* was produced by amplifying an approximately 1.6 kb *ARR5* promoter sequence via PCR using the primers 5′-GCCAAGCTTGGAAACCAATAAAGCATATTTG-3′ and 5′-GCTGTCGACATCAAGAAGAGTAGGATCGTGAC-3′, inserting it into pBI101, and transformation of the construct into Col-0. The *ARR5*_*pro*_*:GUS* and *DR5*_*pro*_*:GUS* constructs had antagonistic expression patterns, with *ARR5*_*pro*_*:GUS* expressed at a relatively high level in the regeneration-competent cells and at an undetectable level in the adventitious root founder cells and the adRP (Fig. 1E). In the adRAM, it was expressed in the root cap and vascular regions but not in the stem cell niche (Fig. 1E). In addition, LOV treatment could lead to the reduced GUS signal in the adRAM (Supplementary Fig. S1B).

The auxin and cytokinin marker lines indicate that auxin and cytokinin likely function antagonistically in different steps and cells during the cell fate transition and cell differentiation associated with adventitious root organogenesis. Briefly, the adventitious root founder cells and the adRP contain a high level of auxin and an undetectable level of cytokinin. In the adRAM, auxin is accumulated in the stem cell niche region, whereas cytokinin is accumulated in the root cap and vasculature.

A modified pseudo-Schiff propidium iodide (mPS-PI) staining method (Truernit et al. 2008; Pi et al. 2015) was used to analyze the newly formed adventitious root tip. The comparison with the wild-type Col-0 revealed defects in cell division and starch grain accumulation in the columella of the root cap as well as an abnormally organized vasculature and ground tissue in *ahk2-2 ahk3-3 ahk4-1* and *arr1-3 arr10-5 arr12-1* (Fig. 1F–H). The stem cell niche marker *WOX5*_*pro*_*:GUS* (Sarkar et al. 2007; Liu et al. 2014) was expressed in a wider region of the adventitious root tip in *arr1-3 arr10-5 arr12-1* than in Col-0 (Fig. 1I, J). The columella stem cell marker J2341 (C24 genetic background) (Pi et al. 2015) was more extensively expressed in the stem cell niche in *ahk2-2 ahk3-3 ahk4-1* (+ / −) and *arr1-3 arr10-5 arr12-1* (+ / −) than in the wild-type C24 (Fig. 1K–M). Furthermore, it was ectopically expressed in the vascular regions and columella of the two mutants, but not in C24 (Fig. 1K–M). These observations suggest that the stem cell niche identity is ectopically maintained in the root cap and the vascular cells if the cytokinin signaling pathway is defective.

In conclusion, the cytokinin signaling pathway contributes to the tissue patterning during the adRP-to-adRAM transition (Fig. 1N). The adRP may be a primitive form of the stem cell niche controlled by a high auxin level (Xu 2018). During the transition from the adRP to the adRAM, the cells in some domains gradually differentiate into functional tissues and eliminate the stem cell niche identity. Cytokinin may be involved in the elimination of the stem cell niche identity and tissue differentiation in those domains via its antagonistic effects on auxin, thereby ensuring the maturation of the adRAM.

### Supplementary Information

**Additional file 1: Supplementary Figure S1.** Analysis of marker genes under LOV treatment.

## Data Availability

The datasets and materials in study are available from the corresponding author upon reasonable request.

## References

[CR1] Bustillo-Avendaño E, Ibáñez S, Sanz O, Barros JAS, Gude I, Perianez-Rodriguez J, Micol JL, del Pozo JC, Moreno-Risueno MA, Pérez-Pérez JM (2018). Regulation of hormonal control, cell reprogramming, and patterning during de novo root organogenesis. Plant Physiol.

[CR2] Crowell DN, Salaz MS (1992). Inhibition of growth of cultured tobacco cells at low concentrations of lovastatin is reversed by cytokinin. Plant Physiol.

[CR3] Della Rovere F, Fattorini L, D’Angeli S, Veloccia A, Falasca G, Altamura MM (2013). Auxin and cytokinin control formation of the quiescent centre in the adventitious root apex of arabidopsis. Ann Botany.

[CR4] Higuchi M, Pischke MS, Mähönen AP, Miyawaki K, Hashimoto Y, Seki M, Kobayashi M, Shinozaki K, Kato T, Tabata S (2004). In planta functions of the Arabidopsis cytokinin receptor family. Proc Natl Acad Sci.

[CR5] Liu J, Sheng L, Xu Y, Li J, Yang Z, Huang H, Xu L (2014). WOX11 and 12 are involved in the first-step cell fate transition during de novo root organogenesis in Arabidopsis. Plant Cell.

[CR6] Mason MG, Mathews DE, Argyros DA, Maxwell BB, Kieber JJ, Alonso JM, Ecker JR, Schaller GE (2005). Multiple type-B response regulators mediate cytokinin signal transduction in Arabidopsis. Plant Cell.

[CR7] Pi L, Aichinger E, van der Graaff E, Llavata-Peris CI, Weijers D, Hennig L, Groot E, Laux T (2015). Organizer-derived WOX5 signal maintains root columella stem cells through chromatin-mediated repression of CDF4 expression. Dev Cell.

[CR8] Sarkar AK, Luijten M, Miyashima S, Lenhard M, Hashimoto T, Nakajima K, Scheres B, Heidstra R, Laux T (2007). Conserved factors regulate signalling in Arabidopsis thaliana shoot and root stem cell organizers. Nature.

[CR9] Truernit E, Bauby H, Dubreucq B, Grandjean O, Runions J, Barthélémy J, Palauqui J-C (2008). High-resolution whole-mount imaging of three-dimensional tissue organization and gene expression enables the study of phloem development and structure in Arabidopsis. Plant Cell.

[CR10] Ulmasov T, Murfett J, Hagen G, Guilfoyle TJ (1997). Aux/IAA proteins repress expression of reporter genes containing natural and highly active synthetic auxin response elements. Plant Cell.

[CR11] Xu L (2018). De novo root regeneration from leaf explants: wounding, auxin, and cell fate transition. Curr Opin Plant Biol.

[CR12] Zhang T, Ge Y, Cai G, Pan X, Xu L (2023). WOX-ARF modules initiate different types of roots. Cell Rep..

