# Supplementary material for: Cytokinin facilitates the patterning of the adventitious root apical meristem from leaf cuttings
Source: Mol Hortic. 2024 Mar 26;4:11. doi: 10.1186/s43897-024-00091-6 (PMC10964562; doi:10.1186/s43897-024-00091-6)
Supplement: Supplementary file 1 — Additional file 1: Supplementary Figure S1. Analysis of marker genes under LOV treatment. [file 43897_2024_91_MOESM1_ESM.pdf]

Supplementary data

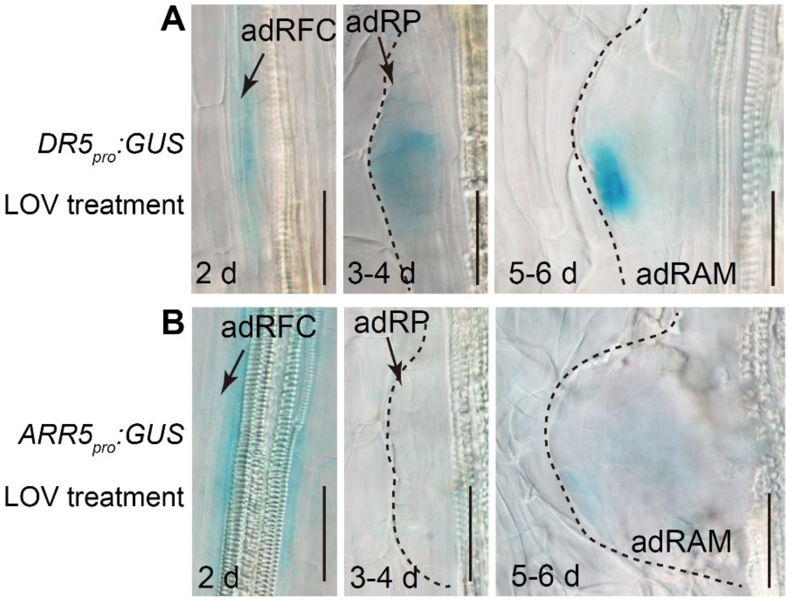

Supplementary Figure S1. Analysis of marker genes under LOV treatment.

(A, B) Expression patterns of *$DR5_{pro}$:GUS* (A) and *$ARR5_{pro}$:GUS* (B) in adventitious root founder cells at 2 days, adRP at 3 to 4 days, and adRAM at 5 to 6 days treated with 30 nM LOV. adRFC, adventitious root founder cells; adRP, adventitious root primordium; adRAM, adventitious root apical meristem.

Scale bars, 50 µm.
